# *VEGF-B*, *VEGF-A*, *FLT-1*, *KDR*, *ERBB2*, *EGFR*, *GRB2*, *RAC1*, *CDH1* and *HYAL-1* Genes Expression Analysis in Canine Mammary Gland Tumors and the Association with Tumor ClinicoPathological Parameters and Dog Breed Assessment

**DOI:** 10.3390/vetsci8100212

**Published:** 2021-09-30

**Authors:** Simona Sakalauskaitė, Violeta Šaltenienė, Darja Nikitina, Rasa Ugenskienė, Vita Riškevičienė, Birutė Karvelienė, Nomeda Juodžiukynienė

**Affiliations:** 1 Department of Veterinary Pathobiology, Faculty of Veterinary Medicine, Veterinary Academy, Lithuanian University of Health Sciences, LT-44307 Kaunas, Lithuania; vita.riskeviciene@lsmuni.lt (V.R.); nomeda.juodziukyniene@lsmuni.lt (N.J.); 2 Laboratory of Clinical and Molecular Gastroenterology, Institute for Digestive Research, Faculty of Medicine, Medical Academy, Lithuanian University of Health Sciences, LT-44307 Kaunas, Lithuania; violeta.salteniene@lsmuni.lt (V.Š.); darja.nikitina@lsmuni.lt (D.N.); 3 Department of Genetics and Molecular Medicine, Faculty of Medicine, Medical Academy, Lithuanian University of Health Sciences, LT-44307 Kaunas, Lithuania; rasa.ugenskiene@lsmuni.lt; 4 Dr. L Kraučeliūnas Small Animal Clinic, Faculty of Veterinary Medicine, Veterinary Academy, Lithuanian University of Health Sciences, LT-44307 Kaunas, Lithuania; birute.karveliene@lsmuni.lt

**Keywords:** canine mammary carcinoma, breed, gene expression, *VEGF-B*, *EGFR*

## Abstract

Canine mammary gland tumors (CMTs) are one of the most prevalent cancers in dogs and a good model for human breast cancer (BC), however gene expression analysis of CMTs is scarce. Although divergence of genes expression has been found in BC of different human races, no such research of different dog’s breeds has been done. The purpose of this study was to investigate expression of the *VEGF-B*, *VEGF-A*, *FLT-1*, *KDR*, *ERBB2*, *EGFR*, *GRB2*, *RAC1*, *CDH1* and *HYAL-1* genes of canine mammary carcinomas, compare the expression levels with clinicopathological parameters and analyze expression disparities between different breeds. Carcinomas and adjacent tissues were collected from female dogs to perform routine histopathology, immunohistochemistry (IHC) and quantitative real-time polymerase chain reaction (qRT-PCR). We found that *VEGF-B* and *EGFR* genes were overexpressed in the mammary gland carcinomas compared to adjacent tissue. *VEGF-B* gene expression had associations with different parameters (tumor size, grade, and absence of metastasis). Furthermore, differences in *VEGF-B*, *FLT1*, *ERBB2*, *GRB2*, *RAC1*, *CDH1* and *HYAL-1* genes expression have been found in different breed dogs (German Shepherd, Yorkshire Terrier) and mixed-breed dogs indicating that a dog’s breed could determine a molecular difference, outcome of cancer and should be accounted as a confounding factor in the future gene expression research.

## 1. Introduction

Canine mammary gland tumors (CMTs) are the most often diagnosed neoplasms in intact female dogs and can make up to approximately 70% of all neoplasms in dogs. Up to 80% of CMTs were found to be malignant, most of them being carcinomas. Morphologically CMTs are very heterogeneous neoplasms and have different clinical behavior [1,2,3]. Different parameters are considered when assessing prognosis of dogs with CMTs. Tumor size, stage, malignancy grade, histological type, lymph node involvement, distant metastasis and proliferation index (PI) are all recognized as prognostic parameters [4,5,6,7,8,9].

CMTs are considered as a good research model for human breast cancer (BC) due to the multiple similarities between them. These tumors develop at an older age, go through an identical course of the disease, clinical stages and invasion to lymph nodes in both humans and dogs [10]. It seems that a few breeds of dogs have an increased risk for different types of cancer, however there is no consensus between researchers upon which breeds have the highest risk to develop CMTs [11,12]. Several breeds (Poodle, Cocker Spaniel, English Springer Spaniel (ESS), German Shepherd, Maltese, Yorkshire Terrier, etc.) were found to have a possible predisposition to CMTs, but this prevalence of predisposition can depend on breed popularity in a particular geographic location [11,13,14]. The exception is ESS from Sweden which was found to have an increased risk for mammary tumors in several studies [11,12]. However, this breed predisposition disparity could be partially attributed to differences in mammary tumors biology, similarly like in a distinction between occurrence and gene expression in BCs of different races. The study of African American (AA) and European American women found 59 differently expressed genes, while another study found differences of multiple positive and negative genes between black and white women [15,16]. To our knowledge, no such studies were performed with CMTs, except an immunohistochemistry (IHC) study which found the similarities between the Shih Tzu breed and AA women because both groups have had the highest prevalence of triple-negative mammary tumors [17].

Genes that had been recognized as critical in the carcinogenesis of humans BCs were found to have the same role in CMTs [10]. Genes encoding vascular endothelial growth factors A and B (*VEGF-A*, *VEGF-B*) and their receptors 1 and 2 (*FLT1*, *KDR*), epidermal growth factor receptor (*EGFR*), tyrosine-protein kinase erbB-2 receptor (*ERBB2/HER2*), Growth Factor Receptor Bound Protein 2 (*GRB2*), Ras-related C3 botulinum toxin substrate 1 precursor (*RAC1*), are all involved in the PI3K/Akt/mTOR (phosphatidyl inositol 3 kinase/kinase B protein/rapamycin target in mammals) signaling pathway which is the hallmark of breast carcinogenesis [18]. The PI3/Akt/mTOR signaling pathway plays an important role in cells proliferation, growth, motility, survival, metabolism and protein synthesis [18,19,20]. 

MAPK (mitogen-activated protein kinase) signaling pathways are also significant pathways incorporating all the above-mentioned genes. They regulate cell proliferation, differentiation and apoptosis, angiogenesis, response to stress and metastasis [21]. Multiple studies have found that activation of PI3/Akt/mTOR and MAPK pathways promote BC cells proliferation and metastasis while suppression of these pathways leads to inhibition of cancer progression [22,23,24,25,26,27]. A recent immunohistochemical study with CMTs confirmed that PI3/Akt/mTOR pathway plays an essential role in tumors aggressiveness in canine cancer too [28]. A study by Uva et al. [29] suggests the striking familiarity in the networks of signaling pathways, including both mentioned above, that the biological and prognostic importance of human BC studies can be carried to CMTs research and vice versa, while these networks and genes have still not been studied so extensively in dogs compared to human.

Cadherin 1 (*CDH1*) gene encoding E-cadherin protein that is responsible for cell–cell adhesion, is a tumor suppressor gene related to the PI3/Akt/mTOR pathway and widely researched in BC with the evidence that downregulation is associated with poor prognostic parameters [28,30,31]. Immunohistochemical studies of E-cadherin variations and association to CMTs clinicopathological parameters have been done in other studies and reduced expression of this membrane protein was found to be associated with tumor aggressiveness and shorter survival time [32,33,34]. Another molecule important for cell adhesion, migration and proliferation is hyaluronic acid generated by hyaluronidase-1 encoded by *HYAL-1* gene [35]. Upregulation of this gene was found to be correlated with malignant behavior in BC and local recurrence in CMTs [36,37,38]. 

In this study we have chosen a panel of cancer related genes (*VEGF-B*, *VEGF-A*, *FLT-1*, *KDR*, *ERBB2*, *EGFR*, *GRB2*, *RAC1*, *CDH1* and *HYAL-1*) in CMTs because of their importance in the signaling pathways of mammary tumors and scarcity of studies done on the molecular level. Therefore, the aim of this study was to investigate the expression of chosen genes in canine mammary carcinomas and their association to clinicopathological parameters. The analysis of gene expression variation according to breed was done, because of the differences noted between dog breeds.

## 2. Materials and Methods

### 2.1. Patients and Samples

The study included 48 female dogs with spontaneously developed mammary gland carcinomas. Tumors and adjacent tissues were collected after surgery as a cancer treatment plan prescribed by their veterinarian and with written consent from the owners. No dogs went into surgery for research or diagnostic purposes or received additional evaluation of metastasis or treatment for cancer. Information from referred veterinarians was collected about dogs age, breed, reproductive status, clinical examination findings and evidence of metastasis. The presence or absence of metastasis was determined according to the customary methods of the clinics. Thoracic radiographs and abdominal ultrasound were done for distant metastasis evaluation. Enlarged lymph nodes were evaluated by cytology after fine needle aspirate and/or resection of the lymph nodes for histopathology assessment. Tumors and adjacent tissues (30–100 mg) were collected into Eppendorf tubes with RNA*later*^®^ solution (Ambion, Austin, TX, USA) and stored at −80°C for quantitative real-time polymerase chain reaction (qRT-PCR). Further, tumors were embedded into paraffin blocks and cut into 6 μm thick sections for conventional histopathology and immunohistochemistry (IHC) to determine proliferation index (PI) by detection of Ki-67.

### 2.2. Histopathology

All tumor biopsies were fixed in 10% buffered formalin and processed and stained according to classical procedures. Histopathological examination was done on hematoxylin–eosin (H&E) stained samples. Histological classification of carcinomas was done according to Goldschmidt et al [39]. The histological grading was performed according to the Elston and Ellis method [40], while the stage was determined in accordance with the modified Tumor-Node-Metastasis (TNM) system for CMTs by World Health Organization [3]. Stage I represent tumors of <3cm in diameter with no involvement of lymph nodes or distant metastasis. Stage II—tumors of 3–5 cm in diameter, while stage III—>5cm with no lymph nodes involvement and distant metastasis in both cases. Stage IV—any size tumor with lymph node involvement without distant metastasis. Stage V—any size tumor with/without lymph node involvement with distant metastasis. In case of multiple tumors from the same dog, the tumor with higher stage and grade was included in the study. Inflammation status and infiltration with white blood cells were assessed. Tumor-infiltrating lymphocytes were counted in three hot spots at ×400 magnification (HPF). Just tumors with no or low/moderate (<50 cells/HPF) lymphocytes infiltration were added to the study. Macrophages were present in 5 tumors.

### 2.3. Immunohistochemistry and PI Evaluation

Immunohistochemistry for Ki-67 was applied for all mammary carcinomas’ samples. Negative control was obtained by omitting primary antibody, while cutaneous tumors were used as positive control. The 6 μm sections of carcinomas were mounted on silane-coated slides. Sections were deparaffinized in xylene, dehydrated with graded ethanol and washed with running and distilled water. Antigen retrieval was done by immersing slides in the Target Retrieval solution (pH 6.0; Dako Denmark A/S, Glostrup, Denmark) at 96 °C for 40 min. EnVision FLEX+, High pH (Link) (Dako Denmark A/S, Glostrup, Denmark) visualization system was used. The washing between every step was done with a Wash Buffer. Endogenous peroxidase was blocked by incubation of sections in Peroxidase–Blocking Reagent for 10 min at room temperature (RT). The samples were coated with primary monoclonal anti-Ki67 antibody (clone MIB-1, Dako Denmark A/S, dilution 1:150, Glostrup, Denmark) for 30 min at room temperature (RT). Later samples were incubated with EnVision FLEX+ Mouse (Linker) and secondary antibody (EnVision /HRP) for 30 min at RT. The color visualization was reached with 3,3-diaminobenzidine tetrahydrochloride (DAB + Chromogen). Samples were counterstained with Mayer′s Hematoxylin Solution (Sigma–Aldrich, Saint Louis, MO, USA), dehydrated, cover-slipped and evaluated by light microscopy (Olympus BX36, Tokyo, Japan). 

The evaluation of Ki-67 was done by counting 1000 cells with immunoreactivity in sample areas representing the hot spots. Immunoreactivity was considered when cells had stained nucleus, irrelevant to its staining intensity. The Ki-67 score (PI) was expressed as the percentage of positively stained cells among the total number of tumor cells in the areas scored.

### 2.4. Tissue sample Preparation and RNA Extraction

Canine mammary gland and adjacent tissue samples were stored in RNA*later* (Ambion, Austin, TX, USA) at +4 °C and 24 h later stored at −80 °C. For RNA extraction tissue sample (50–100 mg) was homogenized in sterile condition using 1 mL TRIzol^TM^ reagent and ceramic beads 2.8 mm “Fisherbrand” in a homogenizer (MagNA lyser, Roche, Indianapolis, IN, USA) at 4000 speeds (1–2 min). Homogenate (0.1–1.4 mL) was transferred to the Phasemaker^TM^ tube with 0.1 ml chloroform and centrifuged at 12,000× *g* at +4 °C. After centrifugation RNA was extracted from an aqueous phase (~100 µL) using TRIzol™ Plus RNA Purification Kit (Invitrogen, Carlsbad, CA, USA) following the manufacturer’s instructions. RNA concentration and purity were determined using a spectrophotometer Nanodrop 2000 (Thermo Scientific, Waltham, MA, USA).

### 2.5. Reverse Transcription and Quantitative Real-time Polymerase Chain Reaction (qRT-PCR)

cDNA for gene expression analysis was synthesized from 2 µg of RNA using a High-Capacity cDNA Reverse Transcription Kit (Applied Biosystems, Carlsbad, CA, USA), according to the manufacturer ‘s protocol. For quantitative RT-PCR, cDNA was diluted to 26 ng per reaction. The expression level of genes were measured using TaqMan^TM^ Gene Assays with FAM dye (*VEGF-B* Cf02721109_u1, *VEGF-A* Cf02674018_m1, *FLT-1* Cf02696454_g1, *KDR* Cf02627749_m1, *ERBB2* Cf02621873_g1, *EGFR* Cf02626541_m1, *GRB2* Cf02667172_m1, *RAC1* Cf02699525_m1, *CDH1* Cf02624268_m1, *HYAL-1* Cf02718719_s1) and TaqMan Universal Master Mix (Applied Biosystems, Carlsbad, CA, USA) on the 7500 Fast Real-Time PCR System (Applied Biosystems, Carlsbad, CA, USA), according to the manufacturer‘s protocol. Gene expression results were calculated using the ΔΔCT method, using *RPL8* (Assay ID: Cf02663822_m1) as the reference gene.

### 2.6. Statistical Analysis

The whole statistical analysis of qPCR data was performed in R software (4.0.4) [41]. Paired *t*-test was used to compare means between tumor and tumor adjacent tissues, when the data was normally distributed, otherwise nonparametric paired samples Wilcoxon test was used. To compare different clinical parameters within tumor or tumor adjacent group unpaired *t*-test was used for parametric data with equal groups variance and unpaired two-sample Wilcoxon test otherwise. Shapiro–Wilk test was used to determine normality. For the correlation analysis, Pearson test was used for parametric data and Spearman test was used for nonparametric data. *p*-values < 0.05 were considered significant.

## 3. Results

### 3.1. Patients and Tumors Characteristics

Mammary gland carcinomas and adjacent normal tissue samples of different breed dogs (*n* = 48) were selected for this study. The breeds included were mixed breed (*n* = 18), German Shepherd (*n* = 11), Yorkshire Terrier (*n* = 5) and other breeds (*n* = 14) (Supplementary Table S1). The average age of dogs was 10.5 ± 2.6 years. Clinicopathological parameters of the tumors are described in Table 1. 

### 3.2. Differentially Expressed Genes in Mammary Carcinoma Tissue of All Breed Dogs

**Figure 1:**
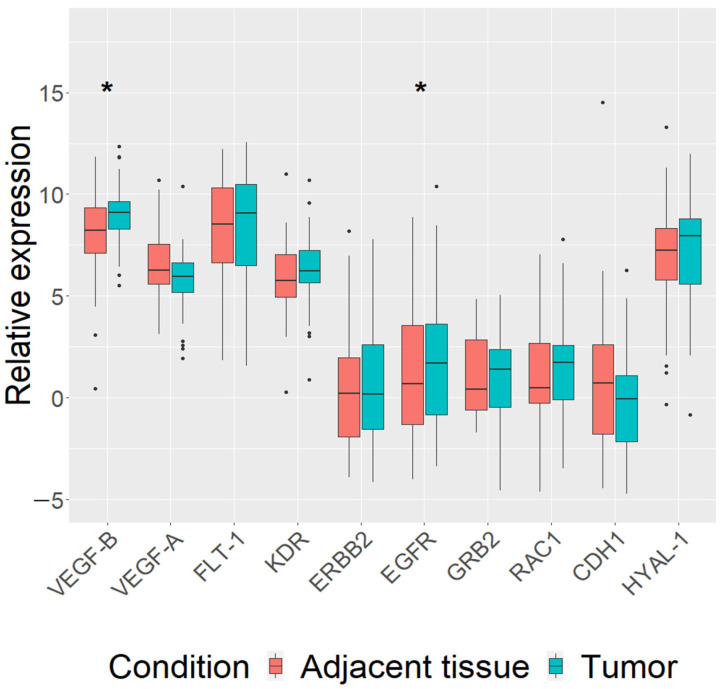
Expression levels of 10 analyzed genes (*VEGF-B*, *VEGF-A*, *FLT-1*, *KDR*, *ERBB2*, *EGFR*, *GRB2*, *RAC1*, *CDH1* and *HYAL-1*) in canine mammary tumors (CMTs) and tumor adjacent tissues in all studied dogs. qRT-PCR data are represented as delta Ct values. Dots indicate outliers. * *p* < 0.05 measured with Wilcoxon or *t*-tests.

First, we analyzed the expression of our chosen genes (VEGF-B, VEGF-A, FLT-1, KDR, ERBB2, EGFR, GRB2, RAC1, CDH1 and HYAL-1) in carcinomas of all dogs regardless of their breed. The expression of *VEGF-B* (log FC = 1.5, *p* = 0.014) and *EGFR* (log FC = 1.6, *p* = 0.017) genes was found to be upregulated in mammary carcinoma tissue (*n* = 48), compared to paired adjacent normal tissue (Figure 1). Analysis including clinicopathological parameters data showed that *VEGF-B* (log FC = 1.5, *p* = 0.013) and *EGFR* (log FC = 1.8, *p* = 0.02) genes were upregulated in mammary carcinoma tumor tissue of dogs’ metastasis-free (*n* = 37) (Supplementary Figure S1). Furthermore, the expression of *VEGF-B* was upregulated (log FC = 2.6, *p* = 0.02) in III stage (≥5 cm) mammary carcinomas (*n* = 24), while the expression of *CDH1* was downregulated (log FC = 3.06, *p* = 0.032) (Supplementary Figure S2). 

**Figure 2:**
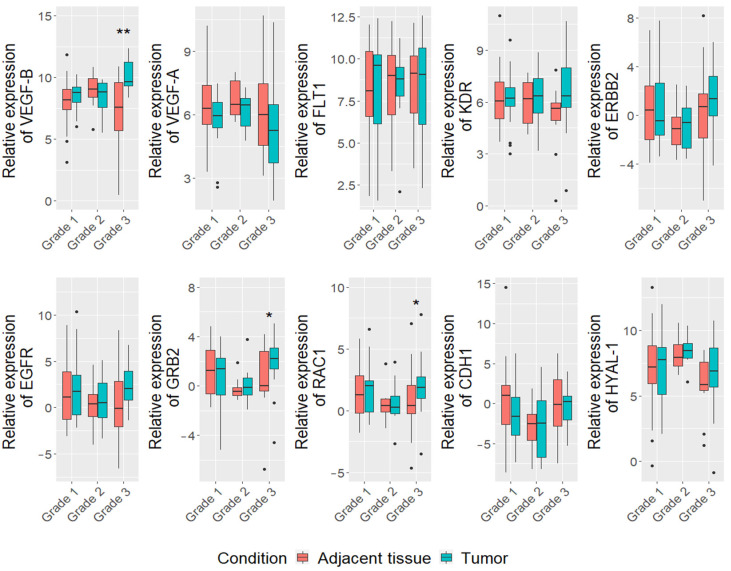
Expression levels of 10 analyzed ) in CMTs and tumor adjacent tissues in different grade carcinomas. Dots indicate outliers. * *p* < 0.05, ** *p* < 0.01 measured with Wilcoxon or *t*-tests.

Regarding tumor grade, the higher expression of *VEGF-B* (log FC = 10.8, *p* = 0.002), *GRB2* (log FC = 2.4, *p* = 0.01) and *RAC1* (log FC = 1.8, *p* = 0.02) genes were detected in mammary carcinoma tumor tissue, compared to adjacent tissue in group of all breed dogs with tumor grade III (*n* = 13) (Figure 2). Furthermore, we found that the expression level of *VEGF-B* gene was higher in the III grade than I grade (*p* = 0.002) and II grade (*p* = 0.009) tumors). Moreover, correlation analysis showed that the expression of *VEGF-B* gene is associated with proliferation index (*r* = 0.39, *p* = 0.005) (Supplementary Figure S3A).

Analyzing histological types, we found an upregulation of *RAC1* levels in carcinoma-mixed type (log FC = 1.8, *p* = 0,027) (Supplementary Figure S4), however there were no significant differences in *VEGF-B* or other genes expressions levels in other carcinoma types.

### 3.3. Gene Expression in Mammary Gland Carcinomas According to the Dog Breed

**Figure 3:**
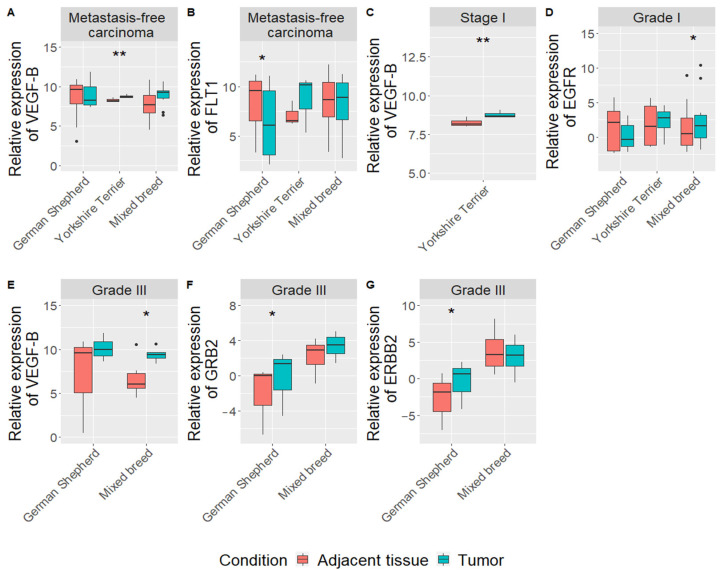
Expression levels of analyzed genes (*VEGF-B*, *FLT-1*, *EGFR*, *GRB2* and *ERBB2*), considering clinical parameters in CMTs and tumor adjacent tissues of different dog breeds. Expression levels of *VEGF-B*, and *FLT* genes in metastasis-free carcinoma samples (**A**,**B**), *VEGF-B* in groups with carcinoma stage I (**C**), *EGFR* in group with grade I (**G**), *VEGF-B*, *GRB2* and *ERBB2* in groups with carcinoma stage III (**E**–G). Dots indicate outliers. * *p* < 0.05, ** *p* < 0.01 measured with Wilcoxon or *t*-tests.

Narrowing of gene expression and clinicopathological data analysis by dog breeds showed some interesting differences between German Shepherd, Yorkshire Terrier and mixed-breed dogs. First, in German Shepherd, we found downregulation of *FLT1* gene (log FC = 6.7, *p* = 0.016) in mammary carcinoma tissue, compared to adjacent tissue in the metastasis-free group (*n* = 8) (Figure 3B). *HYAL-1* gene expression was upregulated (log F = 6.6; *p* = 0.043) in the adjacent tissue, comparing dogs with tumor metastasis (*n* = 3) to dogs without metastasis (*n* = 8) (Supplementary Figure S5). Meanwhile, upregulation of *GRB2* (log FC = 3.9, *p* = 0.016) and *ERBB2* (log FC = 5.6, *p* = 0.026) expression was detected in mammary carcinoma tissue with grade III (*n* = 3) (Figure 3F,G).

In terms of Yorkshire Terrier only *VEGF-B* gene was upregulated in metastasis-free mammary carcinoma tissue, compared to adjacent tissue (log FC = 1.4, *p* = 0.005) (*n* = 5) (Figure 3A) and with tumor stage I (*n* = 5) (log FC = 1.4, *p* = 0.006) (Figure 3C).

In the mixed-breed dog group (*n* = 18), results showed that *EGFR* (log FC = 1,8, *p* = 0.04) and *VEGF-B* (log FC = 14,7, *p* = 0.049) genes were upregulated in mammary carcinoma tissue of tumor grade I and III, respectively (Figure 3D,E). Furthermore, expression of *GRB2* gene is associated with the grade of tumor in mixed breed dogs (*p* = 0.039): the III grade tumors had the higher expression than the I grade tumors. Meanwhile, *EGFR* (*p* = 0.008), *RAC1* (*p* = 0.03) and *VEGF-B* (*p* = 0.0004) genes expression in mammary carcinoma tissue were higher in the high proliferation index group (≥14%) than in low proliferation index group (<14%) (Supplementary Figure S6). Expressions of *FLT1* (*r* = 0.6, *p* = 0.005), *EGFR* (*r* = 0.6, *p* = 0.015) and *VEGF-B* (*r* = 0.8, *p* = 2 x 10^-5^) correlated with proliferation index in mixed breed dogs: increase in gene expression leads to increase in PI (Supplementary Figure S3B–D).

Interestingly, in the initial stage of analysis with all breed dogs we have found only one gene (*RAC1*, *p* = 0.027) which expression was associated with histological type. However, looking at mixed–breed dog group besides *RAC1* gene, we also have found two more genes (*CDH1* and *GRB2*), with changes in their expression level (*p* = 0.019, *p* = 0.002, *p* = 0.012, respectively. Those genes were upregulated in carcinoma-solid compared to carcinoma-simple. 

## 4. Discussion

The frequency in occurrence of canine mammary carcinomas is high, making these tumors a notable part of canine health and a therapeutic challenge. Some clinicopathological parameters are widely recognized as prognostic, which includes tumor stage, histological type and grade of malignancy [42]. However, it is known that the same histological subtype has different phenotypes resulting in different outcomes [43]. Therefore, the aim of this study was to analyze genes (*VEGF-B, VEGF-A, FLT-1, KDR, ERBB2, EGFR, GRB2, RAC1, CDH1* and *HYAL-1*) expression and to evaluate its association with clinicopathological parameters. 

In our study, two genes (*EGFR* and *VEGF-B*) were differentially expressed in the canine mammary tissue compared to the adjacent. *EGFR* was found overexpressed in metastasis-free tumors in all breed dogs. It is known that EGFR is involved in tumors differentiation and proliferation, survival, angiogenesis and metastasis. EGFR protein was found overexpressed in CMTs, but the association with clinicopathological parameters had been contradictory [44]. Increased EGFR expression was associated with a high malignancy grade, a poor clinical stage and large tumor size [44,45]. However, to our knowledge, no studies were conducted measuring the mRNA levels of *EGFR* in CMTs and adjacent tissue, while it is known that there can be divergence between *EGFR* gene and protein expression [46]. Studies in BCs and CMTs have shown increased EGFR expression in mRNA and protein levels in in situ carcinomatous sites relative to invasive areas implying that EGFR plays a more important role in the primary stages of tumor development [46,47]. 

VEGF-A is the main promoter of angiogenesis and mediates its functions mostly through KDR, while VEGF-B binds only to FLT1. VEGF-B role in carcinogenesis is still not completely clear. Primary studies found that VEGF-B promotes angiogenesis, but later studies found the opposing results, while it looks like that this molecule is more important for the survival of certain cell types, including endothelial cells [48,49]. *VEGF-A* RNA levels were found to be upregulated and VEGF-A protein overexpressed in malignant CMTs compared with normal mammary glands [50,51]. *VEGF-A* expression was increased in our study in malignant mammary gland tumors of all breed dogs but not significantly (*p* = 0.06), though it seems to tend to become significant with a larger sample group. This divergence between our and other studies results could be explained by the different choice of samples material: other studies compared CMTs with normal mammary gland tissue of healthy dogs, while we compared the gene expression levels in CMT and adjacent tissue of the same animal. While multiple studies were done in the research of importance of VEGF family members, the knowledge about *VEGF-B* is scarce. *VEGF-B* (together with *VEGF-A*, *FLT1* and *KDR*) was found to be upregulated in invasive ductal carcinoma BC group compared to patients with fibroadenoma, while another study did not find a significant difference in VEGF-B mRNA levels between normal and neoplastic breast [52,53]. To our knowledge, there are no studies that evaluated *VEGF-B* gene expression in CMTs. In our study, *VEGF-B* was not just upregulated in carcinomas compared to adjacent tissue, but was associated with non-metastatic tumors, III stage, and grade tumors and correlated with proliferation index. In contrast, there was no significant association with grade or tumor size in BC, while there was a significant association with positive lymph nodes, suggesting that VEGF-B promotes tumor spread by enhancing metastasis [53]. 

Other genes associated with clinicopathological parameters of all dogs’ CMTs in our study were *RAC1*, *GRB2* and *CDH1*. The *CDH1* gene encodes E-cadherin which maintains cell–cell adhesion; therefore, lack of E-cadherin leads to decreased adhesion between cells and increased tumor invasiveness [31]. *CDH1* was downregulated and associated with large size (>5 cm) of tumor. In BC, reduced E-cadherin expression was associated with larger tumor size, higher grade of malignancy and distant metastasis [54]. Similarly, in CMTs reduced E-cadherin protein expression was associated with tumor size, lymph node metastasis, but not histological grade [32]. A study by Canadas et al. [55] found that different genetic variations of *CDH1* gene were significantly associated with different tumors sizes (≤3 or >3 cm), however, no single nucleotide polymorphisms were associated with lymph node metastasis. Our results showed no association with metastasis, and we see those results of previous studies done with protein expression show variable results, therefore further studies are needed in the expression levels and mutations of the *CDH1* gene in CMTs. 

We found that *GRB2* and *RAC1* gene expression levels were increased in the III grade carcinomas. The *GRB2* gene encodes an adaptor protein which forms complexes with molecules like protein tyrosine kinases and is a key control point in MAPK signaling, while *RAC1* is involved in cell adhesion, motility and proliferation and MAPK signaling [56,57]. Comparative study of pathway expression analysis in human and canine mammary tumors found upregulation of *RAC1* gene expression and downregulation of MAPK signaling pathway in CMTs [29]. Furthermore, *RAC1* had been the only gene associated with histological type analyzing carcinomas of all dogs. Expression of this gene was increased in the carcinoma-mixed type. It is hypothesized that mesenchymal component of the mixed tumors in dogs arise from myoepithelial cells, while *RAC1* is indicated in promotion of epithelial–mesenchymal transition in various human cancers [58,59,60]. Therefore, overexpression of *RAC1* could show its importance in the epithelial transformation to cartilage or bone in the canine mixed tumors.

After dividing dogs into groups according to breed we found upregulation of *RAC1* together with *GRB2* and *CDH1* in carcinoma-solid compared to carcinoma-simple in mixed-breed dogs. In contrast, expression of E-cadherin protein was found to be decreased in the solid carcinoma of dogs in another study [32]. This difference could be because of different molecules measured (RNA versus protein) and because *CDH1* gene is prone to mutations which leads to downregulation of the encoded protein. In our study, we have not analyzed mutations, which will be the next step. On the contrary, oncogene and tumor suppressor *RAC1* is rarely mutated, but upregulation or downregulation themselves leads to enhanced functions of this gene [58]. 

In mixed-breed dogs’ high tumor proliferation index was associated with the overexpression of *RAC1*, *EGFR* and *VEGF-B*, and moderate and strong correlations were found between PI and *FLT1*, *EGFR* and *VEGF-B*. A high PI shows tumors’ ability to grow and metastasize [61], therefore it could be correlated with genes promoting tumors growth. In terms of *FLT1*, it was found to be downregulated in the German Shepherd carcinomas in tumors without metastasis. *FLT1* is thought to be important for angiogenesis, tumor growth and metastasis [62], therefore downregulation could help limit formation of metastasis. Another gene of interest to us was *HYAL-1,* because of the lack of research in CMTs and just several studies in BC. *HYAL-1* expression was significantly increased in the adjacent tissue in the group with metastasis compared to tumor tissue in the group without metastasis. It is known that hyaluronidase (encoded by *HYAL-1*) dissolves hyaluronic acid into small fragments which promotes angiogenesis, possibly leading to increased tumor growth and metastasis [63]. Study in the canine mammary carcinomas did not find any *HYAL-1* gene expression differences in tumor vs normal tissue in the group with metastasis compared to group without metastasis [38]. We observed *HYAL-1* gene expression level changes just in German Shepherds, not in multiple breeds as it was reported in the other study.

A few more interesting differences in gene expression have been found in mixed –breed and German Shepherd groups. Upregulation of *GRB2* gene together with *ERBB2* (*HER2*) was noticed in the III grade tumors of German Shepherd. *GRB2* gene expression was found to be associated with malignancy grade in the mixed-breed group, while *EGFR* and *VEGF-B* were associated with I and III grade tumors. Research in BC showed that GRB2 is a pivotal molecule in tumor growth and downregulation of its expression can lead to inhibition of breast cells with high *EGFR* and *ERBB2* expression levels [63]. HER2 is one of the most researched molecules in BC and it is associated with poor prognosis, poor morphological differentiation and high proliferation rate leading to high grade tumors [64]. IHC studies in CMTs show that HER2 overexpression is associated with high malignancy grade as in our results [65,66]. However, this *HER2* overexpression is seen just in the German Shepherd group, while the mixed-breed dog group has an increase in *EGFR* levels, showing a possible molecular disparity between different breeds therefore leading to different outcomes in the future. Similarly, a recent study in BC showed a similar racial disparity between White and Black women in HR−/HER2+ and HR+/HER2− patients [67]. 

## 5. Conclusions

Our study has shown that *VEGF-B* and *EGFR* genes were overexpressed in the canine mammary gland carcinomas compared to adjacent tissue. Besides, we found an association between *VEGF-B* gene levels and large tumors (III stage), high grade and absence of metastasis at the time of diagnosis, unlike in studies in BC. Therefore, our results warrant deeper studies about *VEGF-B* importance in carcinogenesis in CMTs and possible differences with BC. To our knowledge this is the first research of *VEGF-B*, *GRB2*, *RAC1* and *CDH1* gene expression in CMTs compared to adjacent tissue using quantitative RT-PCR. *GRB2* and *RAC1* have been associated with low proliferation grade. Interestingly, overexpression of *RAC1* was seen in carcinoma-mixed type. Downregulation of *CDH1* has been associated with large tumor size. Further studies would be of interest to investigate functions of *RAC1* and *GRB2*, especially in different histological types of CMTs. Analyzing mammary gland carcinomas of different dog breeds (German Shepherd and Yorkshire Terrier) and mixed–breed dogs, some differences in *VEGF-B*, *FLT1*, *ERBB2*, *GRB2*, *RAC1*, *CDH1* and *HYAL-1* genes expression have been found, possibly showing that some breeds could have a limiting possibility to be used as a good BC model and while analyzing gene expression of canine mammary carcinomas, attention should be given to dog breed as a possible confounding factor. However, breed subgroups in our study were small and the results should be validated in the bigger study.

## Figures and Tables

**Table 1:** Clinicopathological characteristics of the canine mammary tumors (*n* = 48).

Clinicopathological Parameters	Number of Cases
Histological type	
Carcinoma-simple	6
Carcinoma-solid	12
Comedocarcinoma	1
Carcinoma-complex type	11
Carcinoma-mixed type	9
Intraductal papillary carcinoma	3
Squamous cell carcinoma	1
Malignant myoepithelioma	2
Inflammatory carcinoma	3
Grade of malignancy	
Grade I	27
Grade II	8
Grade III	13
Stage	
Stage I	8
Stage II	5
Stage III	24
Stage IV	9
Stage V	2
Metastasis	
Yes	9
No	37
Proliferation index	
Low (<14%)	27
High (≥14%)	21
Inflammation	
Absent	38
Present	10

## Data Availability

The datasets generated in this study are available on request from the corresponding author, as not all data from the study has been published yet.

## Supplementary Materials

The following are available online at https://www.mdpi.com/article/10.3390/vetsci8100212/s1, Table S1. Description of participants and mammary gland carcinomas. Figure S1. *VEGF-B* (**A**) *and EGFR* (**B**) expression comparison between in metastasis-free carcinoma and adjacent tissue of all dogs. Figure S2. *VEGF-B* (**A**) and *CDH1* (**B**) expression comparison between different stages CMTs of all dogs in tumor and adjacent tissue. Figure S3. Correlation analysis between genes expression level and proliferation indices values. Analysis performed: in all breed dogs group of *VEGF-B* gene (**A**), in mixed–breed dogs groups of *FLT1* (**B**), *EGFR* (**C**), and *VEGF-B* (**D**) genes. Figure S4. Comparison of *RAC1* (**A**), *CDH1* (**B**), and *GRB2* (**C**) genes expression level in tumor tissues between dogs with different types of carcinomas. Figure S5. Comparison of *HYAL-1* gene expression level in tumor adjacent tissues between dogs with and without metastasis. Figure S6. Comparison of *EGFR* (**A**), *RAC1* (**B**), and *VEGF-B* (**C**) genes expression level in tumor tissues between dogs with low (<14%) and high (>14%) proliferation index.
