# Supplementary material for: VEGF-B, VEGF-A, FLT-1, KDR, ERBB2, EGFR, GRB2, RAC1, CDH1 and HYAL-1 Genes Expression Analysis in Canine Mammary Gland Tumors and the Association with Tumor ClinicoPathological Parameters and Dog Breed Assessment
Source: Vet Sci. 2021 Sep 30;8(10):212. doi: 10.3390/vetsci8100212 (PMC8537314; doi:10.3390/vetsci8100212)
Supplement: Supplementary file 1 [file vetsci-08-00212-s001.zip › vetsci-1372622-supplementary.pdf]

Supplementary Table S1. Description of participants and mammary gland carcinomas.

Row no.	Breed	Age (years)	Carcinoma type	Malignancy grade	Stage
1.	American Toy Terrier	11	Carcinoma-solid	I	III
2.	Boxer	10	Carcinoma-mixed type	I	III
3.	Dachshund	15	Carcinoma-solid	II	III
4.	Bichon Frise	5	Carcinoma-complex type	I	I
5.	German Shepherd	10	Carcinoma-complex type	III	III
6.	German Shepherd	7	Carcinoma-complex type	III	III
7.	German Shepherd	9	Carcinoma-complex type	II	III
8.	German Shepherd	9	Inflammatory carcinoma	I	III
9.	German Shepherd	10	Carcinoma-complex type	II	IV
10.	German Shepherd	10	Intraductal papillary carcinoma	I	III
11.	German Shepherd	10	Carcinoma-solid	I	IV
12.	German Shepherd	8	Carcinoma-simple	I	III
13.	German Shepherd	10	Carcinoma-simple	I	III
14.	German Shepherd	7	Carcinoma-mixed type	III	IV
15.	German Shepherd	10	Carcinoma-mixed type	I	II
16.	Yorkshire Terrier	7	Inflammatory carcinoma	I	I
17.	Yorkshire Terrier	9	Carcinoma-mixed type	II	IV
18.	Yorkshire Terrier	7	Carcinoma-solid	I	I
19.	Yorkshire Terrier	9	Carcinoma-simple	I	V
20.	Yorkshire Terrier	11	Intraductal papillary carcinoma	I	I
21.	Maltese	10	Carcinoma-mixed type	I	II
22.	Miniature Pinscher	8	Carcinoma-complex type	I	I
23.	Miniature Pinscher	14	Carcinoma-mixed type	III	IV
24.	Mixed breed	11	Malignant myoepithelioma	II	III
25.	Mixed breed	14	Malignant myoepithelioma	I	III
26.	Mixed breed	14	Carcinoma-complex type	I	III
27.	Mixed breed	13	Inflammatory carcinoma	I	III
28.	Mixed breed	3	Carcinoma-solid	I	III
29.	Mixed breed	12	Carcinoma-solid	I	III
30.	Mixed breed	12	Carcinoma-solid	I	IV
31.	Mixed breed	14	Carcinoma-mixed type	I	I
32.	Mixed breed	10	Carcinoma-simple	I	II
33.	Mixed breed	15	Carcinoma-simple	III	III
34.	Mixed breed	12	Carcinoma-solid	III	III
35.	Mixed breed	14	Carcinoma-solid	III	III
36.	Mixed breed	14	Carcinoma-complex type	III	III
37.	Mixed breed	15	Carcinoma-solid	III	IV
38.	Mixed breed	10	Intraductal papillary carcinoma	II	III
39.	Mixed breed	14	Carcinoma-simple	I	IV
40.	Mixed breed	11	Carcinoma-mixed type	I	V
41.	Mixed breed	10	Squamous cell carcinoma	III	III
42.	Newfoundland	13	Carcinoma-solid	III	III
43.	Poodle	12	Carcinoma-mixed type	I	II
44.	Scottish Terrier	9	Carcinoma-complex type	I	I
45.	Siberian Husky	12	Carcinoma-solid	III	III
46.	Siberian Husky	10	Carcinoma-complex type	II	II
47.	Vizsla	7	Comedocarcinoma	I	IV
48.	West Highland White Terrier	10	Carcinoma-complex type	III	I

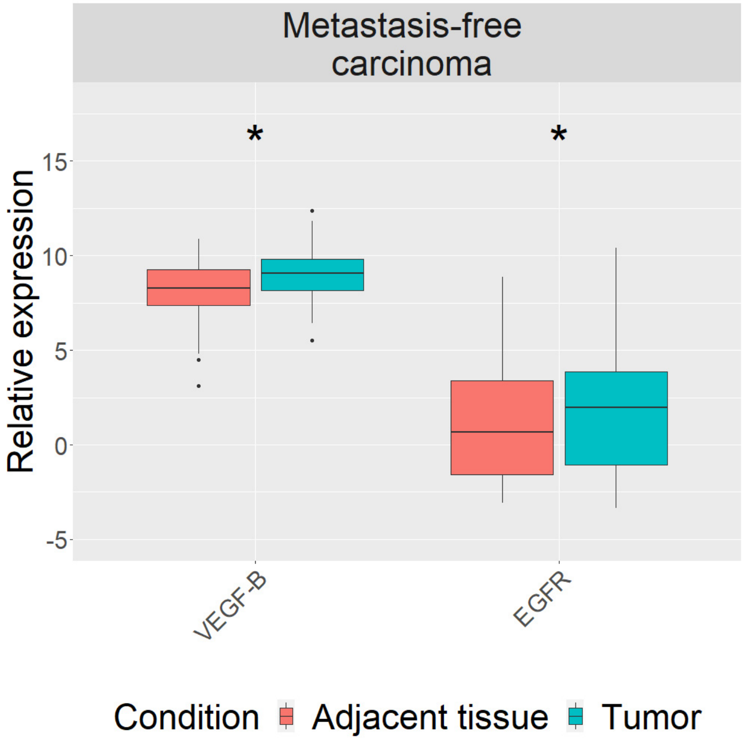

Supplementary Figure S1. *VEGF-B* (**A**) and *EGFR* (**B**) expression comparison between in metastasis-free carcinoma and adjacent tissue of all dogs. Dots indicate outliers. *– $p < 0.05$ measured with Wilcoxon or t-tests.

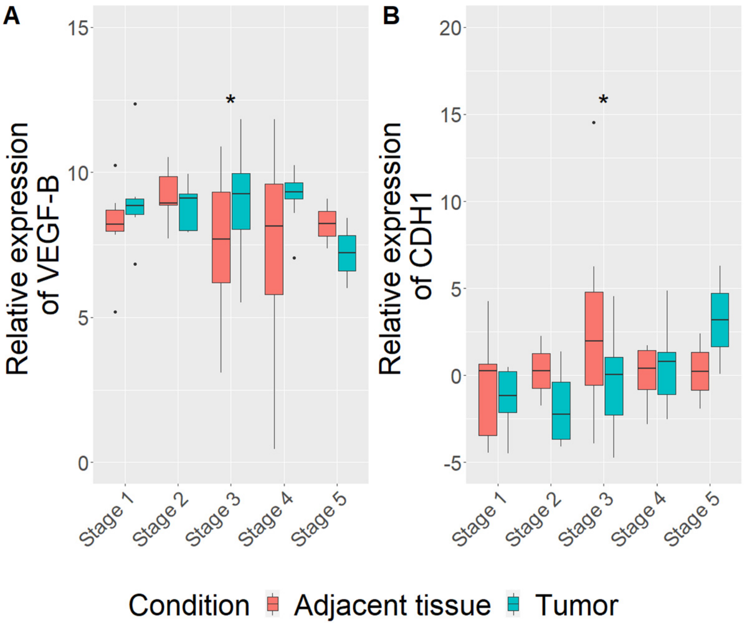

Supplementary Figure S2. *VEGF-B* (**A**) and *CDH1* (**B**) expression comparison between different stages CMTs of all dogs in tumor and adjacent tissue. Dots indicate outliers. *– $p < 0.05$ measured with Wilcoxon or t-tests.

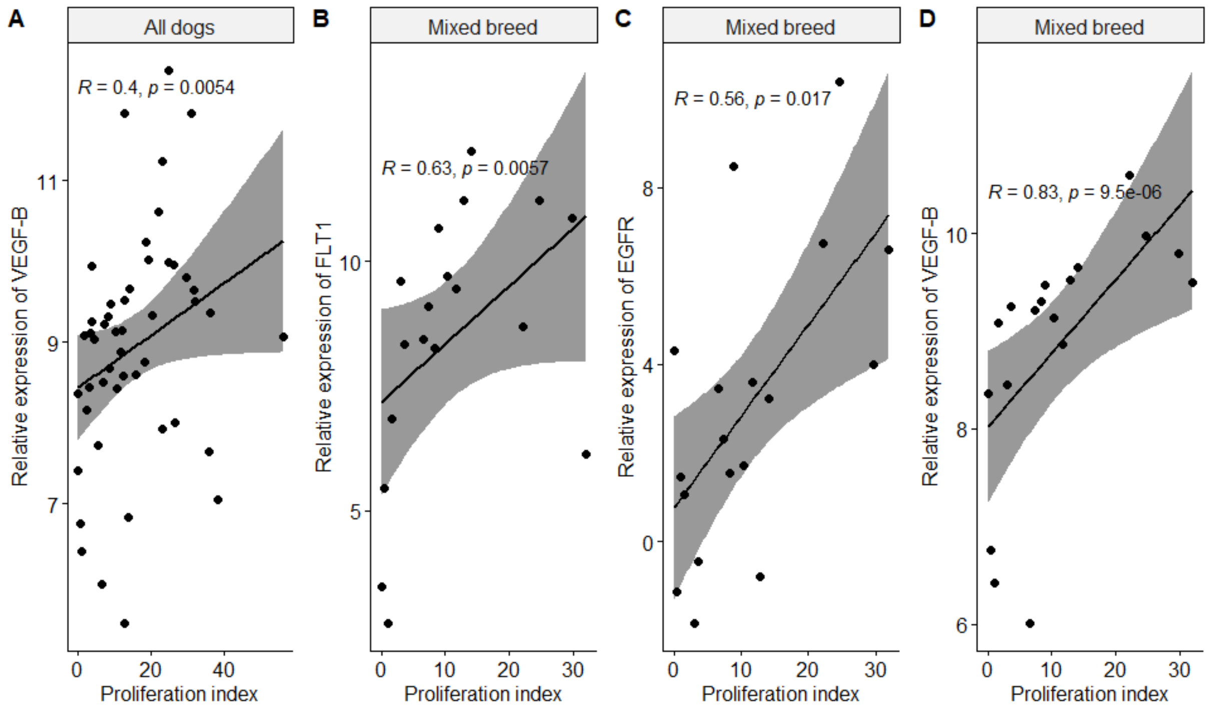

Supplementary Figure S3. Correlation analysis between genes expression level and proliferation indices values. Analysis performed: in all breed dogs group of *VEGF-B* gene (**A**), in mixed–breed dogs groups of *FLT1* (**B**), *EGFR* (**C**), and *VEGF-B* (**D**) genes.

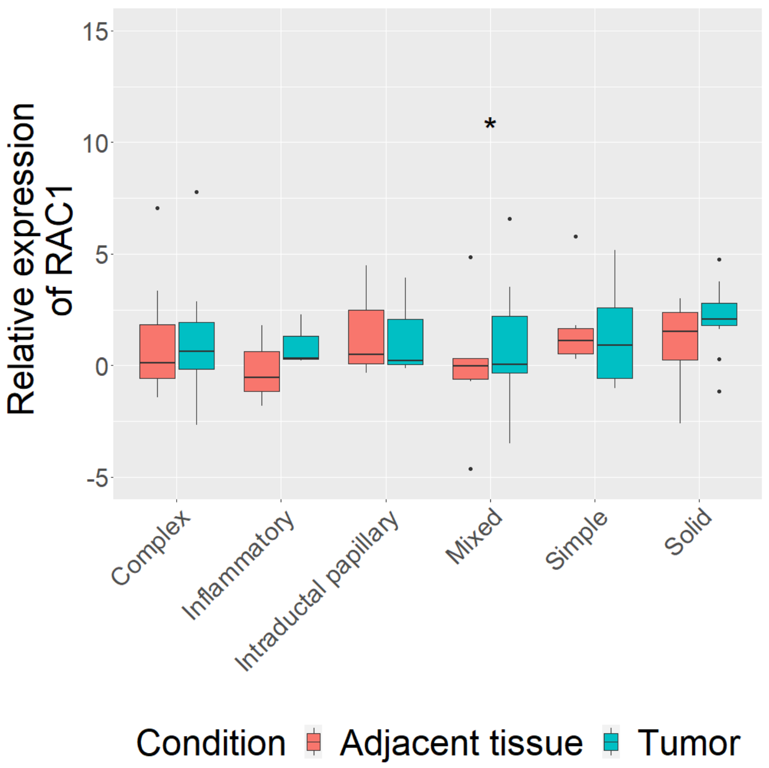

Supplementary Figure S4. Comparison of *RAC1* gene expression level in tumor tissues between dogs with different types of carcinomas. Dots indicate outliers. *– $p < 0.05$ measured with Wilcoxon or T tests.

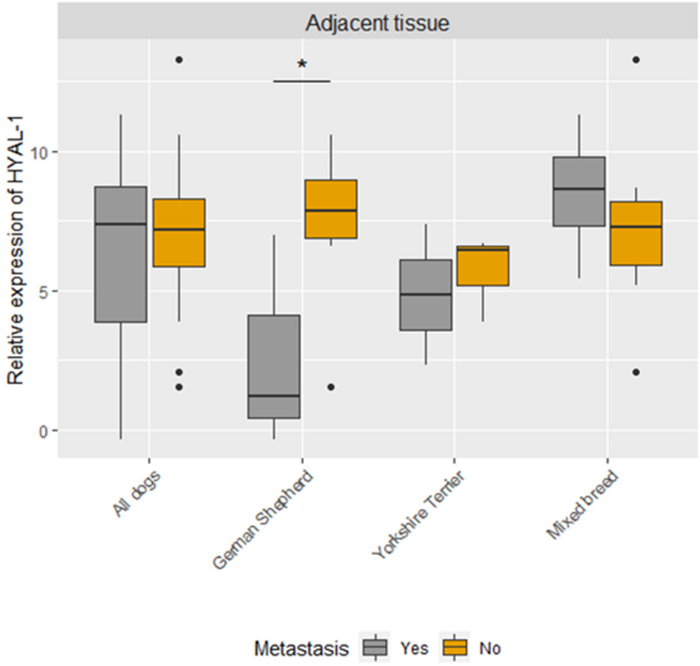

Supplementary Figure S5. Comparison of *HYAL-1* gene expression level in tumor adjacent tissues between dogs with and without metastasis. Dots indicate outliers. *– $p < 0.05$ measured with Wilcoxon or T tests.

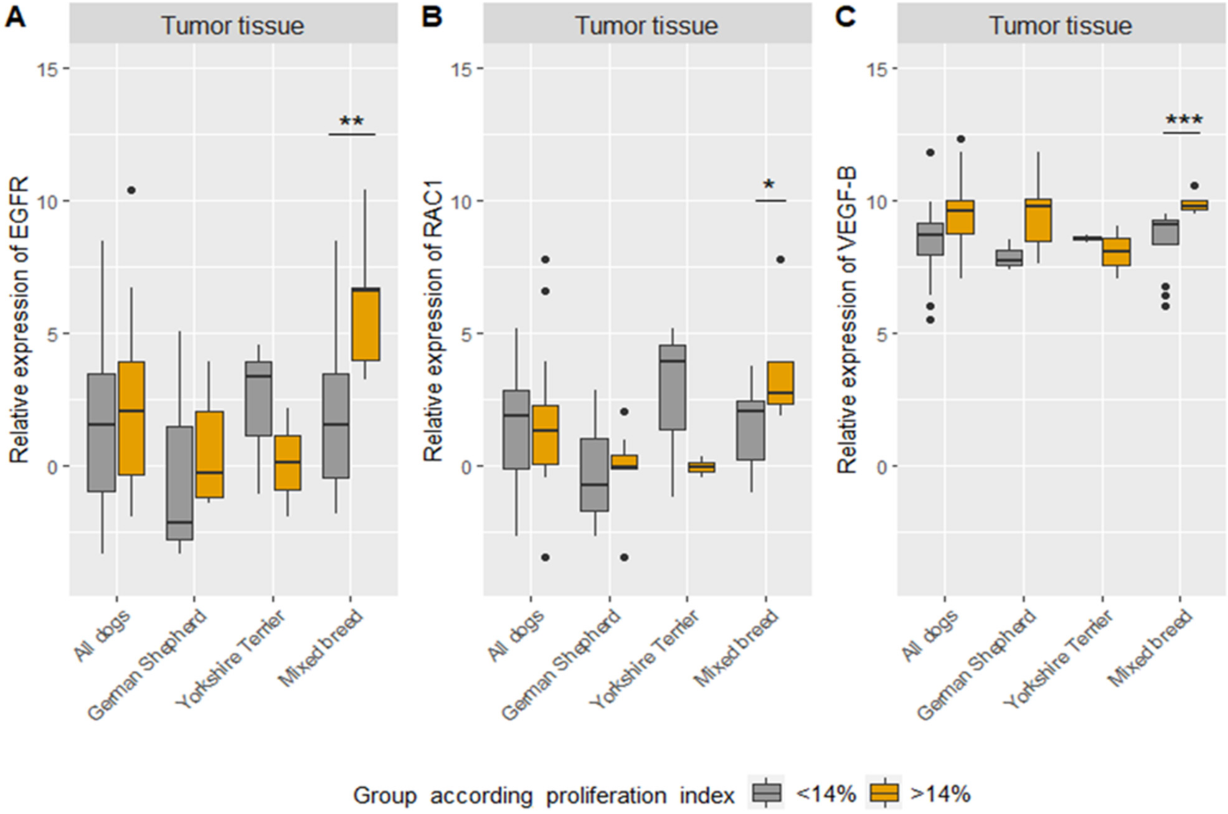

Supplementary Figure S6. Comparison of *EGFR* (**A**), *RAC1* (**B**), and *VEGF-B* (**C**) genes expression level in tumor tissues between dogs with low (<14%) and high (>14%) proliferation index. Dots indicate outliers. *– $p < 0.05$, **— $p < 0.01$, ***– $p < 0.001$ measured with Wilcoxon or t-tests.
